# Combined Effects of Smartphone Overdependence and Stress on Depression and Suicide-Related Behaviors among High School Students

**DOI:** 10.3390/healthcare10091671

**Published:** 2022-09-01

**Authors:** Hyesun Kim

**Affiliations:** Department of Nursing, Hyejeon College, Hongseong 32244, Korea; twins815@hanmail.net

**Keywords:** adolescent, technology addiction, stress, depression, suicide

## Abstract

This study aimed to identify smartphone overdependency and stress’ combined effects on depression and suicide-related behaviors, such as suicidal ideation, plans, and attempts, among Korean high school students. Cross-sectional secondary data analysis was conducted using data from the 2020 Korea Youth Risk Behavior Web-based Survey. This study included 25,987 high school students. Data were analyzed using descriptive analysis, Rao-Scott chi-square test, and logistic regression based on a complex sample design. Regardless of smartphone overdependence, some stress and high stress were associated with higher depression than no stress and no smartphone overdependence. Furthermore, regardless of smartphone overdependence, some stress and high stress were associated with greater depression and suicidal ideation than no stress and no smartphone overdependence. However, only high stress was associated with suicide plans and attempts. Additionally, stress with smartphone overdependence increased the risk of depression and suicide-related behaviors, whereas the absence of stress did not significantly affect depression and suicide-related behaviors. Therefore, to prevent depression and suicide-related behaviors among high school students, continuous monitoring of and interventions to reduce stress levels should be prioritized. Moreover, as stress combined with smartphone overdependence increases the likelihood of depression and suicide-related behaviors, educational programs to prevent smartphone overdependence should be developed.

## 1. Introduction

Depression and suicide are major public health concerns among adolescents worldwide. The suicide rate among adolescents in Korea was 5.9 per 100,000 as of 2019, which was a 1.4-fold increase from 4.2 in 2015 [1], and depression among adolescents increased from 23.6% in 2015 to 28.2% in 2019 [2]. This is an urgent issue, as the number one cause of death among adolescents in Korea is suicide [1]. Recently, the “Corona Blue” phenomenon, indicating depression during the COVID-19 pandemic, drew attention in Korea, as depression increased due to the decrease in social activities caused by the COVID-19 pandemic [3]. Depression in adolescents may become chronic throughout life, leading to a variety of physical and mental health problems, including suicidal ideation, plans, and attempts [4,5]. In addition, although adolescence is a time when sensitivity to mental health problems, such as depression, increases due to adolescents’ irritability and frequent mood reactivity, it is easier to overlook mental health problems than in adulthood [5,6]. Therefore, as mental health problems of adolescents are being emphasized, effective interventions to prevent depression and suicide-related behaviors are urgently needed.

Smartphone overdependence is a problem that manifests as decreased self-control of smartphone use [7]. It is often used interchangeably with the term “smartphone addiction” [7]. Adolescents’ use of smartphones is continuously increasing because they can easily access various activities, such as Internet, social media, and online games [8,9]. However, in adolescence, the cortex responsible for self-control is still immature [5], and the characteristics of adolescence, such as low self-control, impulsivity, emotional imbalance, and diverse and intense sensations, may create greater vulnerability to addictive behaviors, such as smartphone overdependence [8,10]. Recently, as social activities have decreased due to COVID-19, and the time spent using smartphones has increased, the risk of adolescents’ excessive dependence on smartphones has risen from 30.2% in 2019 to 37.0% in 2021, when COVID-19 was prevalent, showing the highest smartphones dependence among all ages [7]. According to a recent meta-analysis, adolescents’ smartphone overdependence causes not only physical symptoms, such as headaches and vision disturbance, but also various mental health problems, such as depression, suicidal ideation, anxiety, obsessive-compulsive symptoms, impulsiveness, sleep disturbance, aggression, concentration problems, and attention deficit hyperactivity disorder [11]. Kim et al. reported that depression increased by 1.13-fold, suicidal ideation increased by 1.14-fold, and suicide attempts increased by 1.3-fold when smartphone usage time increased among adolescents [12]. Therefore, smartphone overdependence is considered a high-risk factor for depression- and suicide-related behaviors in adolescents.

Stress is considered an important factor in the development and recurrence of various behavioral addictions, including gambling, alcoholism, and drug addiction [13]. Therefore, it is highly associated with smartphone overdependence and may lead to suicide-related behaviors, as it is the strongest risk factor for depression [5,13]. According to a previous study, when faced with stress, smartphone use increases as a coping mechanism to reduce emotional tension or avoid psychological distress [14]. It has been reported that high stress is 4.73-fold more likely to lead to smartphone overdependence compared to low stress [14]. In particular, adolescents are highly likely to depend on smartphones, as their coping mechanisms are poor despite being continuously exposed to various stressors [14]. Therefore, as smartphone overdependence and stress can coexist in a significant relationship, the individual and combined effects of smartphone overdependence and stress on depression and suicide-related behaviors in adolescents are expected to be different. However, previous studies focused only on individual effects, such as the effects of stress on suicide-related behaviors [15] and the effects of smartphone overdependence on depression and suicide-related behaviors [8,12]. Therefore, the combined effects of smartphone overdependence and stress on depression and suicide-related behaviors in adolescents merit investigation. In particular, high school students experience considerable stress as they face rapid physical, emotional, cognitive, and sexual changes and various challenges in performing social roles [9,16]. In addition, smartphone overdependence tends to increase over time [8]; therefore, this study focused on high school students.

In the field of psychiatry, the biopsychosocial model proposed by Engel provides a framework for identifying the effects of various covariates, including biological (e.g., genetic or physiological factors and biological processes), social (e.g., culture, environmental factors, socioeconomic status, and social relationship), and psychosocial factors (e.g., health behaviors and beliefs), on mental health problems, such as depression [17,18]. Based on Engel’s biopsychosocial model, in this study, the biological factors are grade and gender, the social factors are residential area, socioeconomic status, and academic achievement, and the psychosocial factors are perceived health status, smoking, drinking, substance abuse, sexual intercourse, physical activity, and sleep quality—these variables were considered as the covariates. Therefore, the effects of the individual and combined effects of smartphone overdependence and stress on depression and suicide-related behavior was confirmed by controlling these factors.

## 2. Materials and Methods

### 2.1. Design and Participants

This cross-sectional study analyzed secondary data from the 16th Korea Youth Risk Behavior Web-based Survey (KYRBWS) conducted in 2020. The KYRBWS, which has been conducted annually since 2005, is a nationwide survey that is conducted with government approval to gather statistical data on the health behaviors of Korean adolescents. Sampling was conducted using population stratification, sampling distribution, and stratified cluster sampling methods, and weights were assigned to represent high school students nationwide. The KYRBWS is an online survey that has been conducted since 2005 with middle and high school students to identify the health behaviors, such as smoking, drinking, diet habits, and physical activity, of Korean adolescents. The 16th KYRBWS included 793 schools nationwide with a total of 54,948 participants. Of which, after excluding 28,961 middle school students, 25,987 high school students were included in this study.

### 2.2. Measurements

#### 2.2.1. Smartphone Overdependence

Smartphone overdependence was measured using the Smartphone Overdependence Scale developed in 2016 by the National Information Society Agency (NIA) [7]. It consists of 10 items rated on a 4-point scale. According to the cutoff point specified by the NIA’s Smartphone Overdependence Scale [7], scores of 23–30 indicate potential risk for smartphone overdependence, and scores of 31 or higher indicate high risk for smartphone overdependence. In this study, scores of 23 or higher were considered smartphone overdependence [7].

#### 2.2.2. Stress

Stress was measured using a question on the level of stress in daily life, and responses were categorized as high stress, some, and no stress.

#### 2.2.3. Outcome Variable

##### Depression

Depression was measured using the following question: “During the past 12 months, have you ever felt sad or hopeless enough to stop your daily life for two weeks?” The responses were divided into “yes” or “no” on the original response scale.

##### Suicide-Related Behaviors

Suicidal ideation was assessed using the following question: “In the past 12 months, have you seriously considered suicide?” Suicide plans were measured using the following question: “In the last 12 months, have you made any specific plans to commit suicide?” Suicide attempts were assessed using the following question: “In the past 12 months, have you attempted suicide?” All responses were classified into “yes” or “no” on the original response scale.

#### 2.2.4. Covariates

##### Biological Factors

Grades and genders were used, and grades were divided into three categories (1st, 2nd, and 3rd).

##### Social Factors

The residential area was divided into three categories: metropolis, middle-sized cities, and rural areas. The economic status of the household was classified into high, middle, and low. Academic performance was classified into high, middle, and “low”.

##### Psychosocial Factors

Perceived health status was classified into three categories: healthy, fair, and unhealthy. Smoking and drinking were measured using the frequency of use over the past 30 days, and more than one day was considered as having experienced smoking or drinking. Drug use was measured using questions on whether drugs or substances were habitually used other than for treatment purposes, and responses were classified as “yes” or “no” on the original response scale. Sexual intercourse was evaluated using a question on whether sexual intercourse was experienced, and responses were classified as “yes” or “no” on the original response scale. Physical activity was classified as sufficient if vigorous or moderate–vigorous intensity physical activity was performed for more than 3 days in the last 7 days, and less than 3 days was classified as insufficient [19,20]. As a measure of sleep quality, a question on whether sleep was sufficient to relieve fatigue during the past week was used, and responses were classified as good, moderate, or poor.

### 2.3. Data Analysis

The KYRBWS was sampled using population stratification, sample distribution, and stratified cluster extraction. The data were analyzed using a complex sample analysis module that considers strata, clusters, and weights. Data analysis was performed using IBM SPSS 25.0 (IBM, Armonk, New York, NY, USA). Descriptive statistics were used to analyze the participants’ demographic characteristics, smartphone overdependence, stress, depression, suicidal ideation, suicidal plans, and suicide attempts. The differences in depression, suicidal ideation, suicidal plans, and suicide attempts according to the participants’ sociodemographic characteristics, smartphone overdependence, and stress were analyzed using the Rao-Scott χ^2^ test. Logistic regression analysis was performed to identify the individual and combined effects of smartphone overdependence and stress on depression, suicidal ideation, suicide plans, and suicide attempts. In the Rao-Scott χ^2^ test, statistically significant variables (*p* < 0.05) were included as covariates in the logistic regression analysis.

## 3. Results

### 3.1. Characteristics of Smartphone Overdependence, Stress, Depression, and Suicide-Related Behaviors among Participants

General characteristics, smartphone overdependence, stress, depression, and suicide-related behaviors of the participants are described in Table 1. Of the 25,987 participants, 27.3% exhibited smartphone overdependence and 37.9% answered that they usually felt high stress. In the past 12 months, 27.4% of the respondents said they had depression, 11.5% had suicidal ideation, 3.4% had suicidal plans, and 2.0% had attempted suicide.

### 3.2. Differences in the Depression and Suicide-Related Behaviors According to General Characteristics, Smartphone Overdependence, and Stress

Table 2 shows the results for differences in depression- and suicide-related behaviors based on general characteristics, smartphone overdependence, and stress. Depression, suicidal ideation, and suicidal plans based on general characteristics differed by grade, gender, economic status of household, academic achievement, perceived health status, smoking, drinking, drug use, sexual intercourse, and quality of sleep. Suicidal attempts differed by grade, sex, residence area, household economic status, academic achievement, perceived health status, smoking, drinking, drug use, sexual intercourse, physical activity, and quality of sleep. In addition, depression and suicide-related behaviors were more frequent with smartphone overdependence or stress.

### 3.3. Individual Effects of Smartphone Overdependence and Stress on Depression and Suicide-Related Behaviors

Table 3 presents the individual effects of smartphone overdependence and stress on depression and suicide-related behaviors. Smartphone overdependence was associated with an increase in depression (adjusted odds ratio (AOR):1.40, 95% confidence interval (CI):1.31–1.49, *p* < 0.001), suicidal ideation (AOR:1.44, 95% CI: 1.32–1.57, *p* < 0.001), and suicidal plans (AOR:1.21, 95% CI: 1.05–1.39, *p* = 0.009). Some stress was associated with an increase in depression (AOR:2.72, 95% CI: 2.44–3.05, *p* < 0.001) and suicidal ideation (AOR:2.42, 95% CI: 1.94–3.03, *p* < 0.001), and high stress was associated with depression (AOR:8.49, 95% CI: 7.53–9.58, *p* < 0.001), suicidal ideation (AOR:9.84, 95% CI: 7.93–12.21, *p* < 0.001), suicidal plans (AOR:4.98, 95% CI: 3.56–6.97, *p* < 0.001), and suicidal attempts (AOR:4.87, 95% CI: 2.94–8.06, *p* < 0.001).

### 3.4. Combined Effects of Smartphone Overdependence and Stress on Depression and Suicide-Related Behaviors

Table 4 shows the combined effects of smartphone overdependence and stress on depression- and suicide-related behaviors. The risk of depression and suicidal ideation increased by 2.56 times (95% CI: 2.27–2.88, *p* < 0.001) and suicidal ideation by 2.10 times (95% CI: 1.64–2.68, *p* < 0.001) with some stress in the absence of smartphone overdependence compared to no smartphone overdependence and no stress. The risk of depression increased by 3.71 times (95% CI: 3.23–4.27, *p* < 0.001) and of suicidal ideation by 3.33 times (95% CI: 2.60–4.25, *p* < 0.001) with some stress and smartphone overdependence.

The risk of depression increased by 8.06 times (95% CI: 7.08–9.17, *p* < 0.001), of suicidal ideation by 8.85 times (95% CI:7.03–11.14, *p* < 0.001), of suicidal plans by 4.36 times (95% CI: 3.01–6.31, *p* < 0.001), and the risk of suicide attempts by 5.33 times (95% CI: 3.00–9.45, *p* < 0.001) with high stress and no smartphone overdependence. The risk of depression increased by 11.29 times (95% CI: 9.84–12.96, *p* < 0.001), of suicidal ideation by 12.58 times (95% CI: 9.97–15.88, *p* < 0.001), of suicidal planning by 5.59 times (95% CI: 3.83–8.14, *p* < 0.001), and of suicide attempt by 5.47 times (95% CI: 3.09–9.68, *p* < 0.001) with high stress and smartphone overdependence. However, smartphone overdependence with no stress was not associated with an increased risk of depression or suicidal behaviors.

## 4. Discussion

This study identified the individual and combined effects of smartphone overdependence and stress on depression- and suicide-related behaviors among high school students using data from the 16th KYRBWS. Regarding the individual effects, it was found that high school students’ smartphone overdependence had significant effects on depression, suicidal ideation, and suicide plans. In addition, some stress had a significant effect on depression and suicidal ideation, and high stress had a significant effect on depression, suicidal ideation, suicide plans, and suicide attempts. Regarding, the combined effects, regardless of whether smartphone overdependence was present, some stress was associated with an increase in depression and suicidal ideation, and high stress was associated with an increase in suicidal ideation, suicide plans, and suicide attempts. These results suggest that stress is an important factor in depression and suicide-related behaviors among high school students. According to previous studies, depression was 3.5 times higher in the high- than low-stress group [16], suicidal ideation was 7.3 times higher, suicide plan by 6.2 times, and suicide attempts were 3.6 times in the high stress group [15].

The high school years are a transitional period of growth and development from childhood to adulthood. They are emotionally unstable and under significant stress due to the burden of preparation and developmental tasks for becoming an adult, such as career choice, self-concept construction, and personality maturation [21]. In particular, the prefrontal cortex and amygdala develop continuously during this period, making them more sensitive and impulsive to stress [22]. However, they are more vulnerable to depression or suicide-related behaviors, as they do not have sufficient free time to relieve stress or have inadequate coping mechanisms to deal with stress [21]. According to a qualitative study, adolescents who experienced depression felt stress due to conflict with parents, academic achievement, and peer relationships [23], and approximately 80% of depressed adolescents said that stress preceded them [24]. Furthermore, impulsive and aggressive behaviors in adolescence are known to constitute a strong risk factor for suicide attempts [25,26], and they may choose to commit suicide impulsively as a way to overcome difficult situations instead of seeking help when they are experiencing stress beyond their control [27]. According to Jung [28], students who attempted suicide were under extreme stress from their parents or interpersonal relationships before attempting suicide. Therefore, stress is a major influencing factor affecting depression and suicidal behavior.

In addition, smartphone overdependence was a variable that further increased the risk of depression and suicidal behavior in the presence of stress. From a neurophysiological point of view, the visual stimulation of a smartphone causes functional abnormalities in the frontal lobe of the brain, increasing the problems of thinking, judgment, and impulse control [9] and reinforcing negative thoughts and behaviors [29]. Furthermore, when they are overly dependent on their smartphones and under significant stress, cognitive thinking and judgment are limited [30], which can increase psychological distress and lead to depression [9]. Therefore, the risk of developing mental health problems increases when smartphone overdependence is combined with stress. Thus, for high school students who are frequently under stress, interventions, such as monitoring their stress levels and the time spent on their smartphones as well as limiting the time on their smartphones, are needed.

However, contrary to our findings, there is some evidence that suggests that depressive symptoms can lead to smartphone overdependence. Smartphones can be used as a coping mechanism to alleviate negative emotions such as low self-esteem, anxiety, and depression, and as a means of satisfying psychological needs [31,32]. In particular, people with depression are more likely to use smartphones to alleviate their symptoms, and because they have low impulse control, they have difficulty managing their smartphone use, thereby, increasing the risk of smartphone overdependence [32]. Heffer and colleagues’ longitudinal study demonstrated that depression preceded the increase in smartphone use [33]. However, Elhai and colleagues reported that excessive smartphone use in people with depression may lead to an increase in depression, suggesting a bidirectional relationship [31]. Therefore, further research is needed to clarify the direction of the relationship between these two variables.

In addition, at a low level of stress, even with smartphone overdependence, there was no association with suicide plans or suicide attempts. This suggests that even if smartphone overdependence and stress coexist, suicide-related behaviors are induced differently, depending on the level of stress. This means that low stress is insufficient to develop suicidal ideation into suicide plans or attempts; however, high stress that is difficult to handle can lead to suicide plans or attempts. Previous studies revealed that the severity of suicidal ideation increased as the level of stress and depression increased [34], and the suicide rate was highest during the test period under the highest academic stress [35]. However, as suicidal ideation, suicidal plans, and suicide attempts are continuous processes, and suicidal ideation precedes suicide planning and suicide attempts, it is an important index for evaluating suicide risk. Therefore, there is a need to continuously monitor the stress level of high school students, recognize the early signs of suicidal ideation, and investigate the inherent risk factors that change from suicidal ideation to suicidal plans and attempts. Based on these results, practitioners need to provide interventions to help improve the ability to cope with problematic situations and self-control, and thereby, improve high school students’ emotional and psychological health levels and reduce their stress levels. Additionally, there is a need to develop and provide programs that include specialists who can screen high school students for smartphone overdependence, and provide them with relevant strategies to help prevent and treat it.

This study had some limitations. First, as this was a cross-sectional study using secondary data, caution is needed when interpreting causal relationships between variables. Second, this study classified stress variables according to stress level, and the factors or frequency of stress were not considered. Third, as the questionnaire was self-reported by high school students, there may be a regression bias. Fourth, coping mechanisms for stress, social support levels, and individual resilience were not considered. Nevertheless, the significance of this study is that it is highly representative due to using the KYRBWS, which contains representative data of high school students in Korea. Second, to the best of my knowledge, this is the first study to investigate the individual and complex effects of smartphone overdependence and stress on depression- and suicide-related behaviors among high school students. The results of this study can be used as evidence and educational material for developing programs to reduce depression and suicide-related behaviors among high school students.

## 5. Conclusions

This study confirmed the combined effect of smartphone overdependence and stress on depression and suicide-related behaviors among high school students. This study found that some stress had a significant effect on depression and suicidal ideation, regardless of whether smartphone overdependence was present, and that high stress had a significant effect on depression, suicidal ideation, suicidal plans, and suicide attempts. Therefore, efforts should be made to explore various factors affecting high school students’ stress and develop strategies focusing on stress protection factors, such as enhancing self-esteem, resilience, ability to cope with stress, and forming social networks.

Future studies should examine the relationship between depression and suicide-related behaviors by subdividing the causes of stress, such as academic, interpersonal, and post-traumatic stress. Moreover, a longitudinal study on the combined effects of smartphone overdependence and stress on depression and suicide-related behaviors should be conducted in the future.

## Figures and Tables

**Table 1:** Characteristics of smartphone overdependence, stress, depression, suicide-related behaviors, and covariates.

Variables	Categories	*n* (%)
Independent variables		
Smartphone overdependence	Yes	6910 (27.3)
	No	19,077 (72.7)
Stress	High	9763 (37.9)
	Some	11,321 (43.6)
	No	4903 (18.6)
Dependent variables		
Depression	Yes	7100 (27.4)
	No	18,887 (72.6)
Suicidal ideation	Yes	2966 (11.5)
	No	23,021 (88.5)
Suicidal plans	Yes	892 (3.4)
	No	25,095 (96.6)
Suicidal attempts	Yes	529 (2.0)
	No	25,458 (98.0)
Covariates		
Biological factors		
Grade	1st	8907 (33.5)
	2nd	8907 (33.8)
	3rd	8173 (32.7)
Gender	Girls	12,464 (47.9)
	Boys	13,523 (52.1)
Social factors		
Residence area	Metropolis	13,127 (51.1)
	Middle-sized cities	11,264 (44.5)
	Rural area	1596 (4.4)
Socioeconomic status of household	High	8701 (34.3)
	Middle	13,102 (50.3)
	Low	4184 (15.4)
Academic performance	High	7730 (29.6)
	Middle	8147 (31.4)
	Low	10,110 (38.9)
Psychosocial factors		
Perceived health status	Unhealthy	2406 (9.4)
	Fair	5901 (22.9)
	Healthy	17,680 (67.6)
Smoking	Yes	2082 (7.7)
Drinking	No	23,905 (92.3)
	Yes	4272 (15.9)
	No	21,715 (84.1)
Drug use	Yes	239 (1.0)
	No	25,748 (99.0)
Sexual intercourse	Yes	1933 (7.3)
	No	24,054 (92.7)
Physical activity	Sufficient	8902 (33.5)
	Insufficient	17,085 (66.5)
Quality of sleep	Good	6471 (25.0)
	Moderate	8720 (33.3)
	Poor	10,796 (41.7)

Note. Values are presented as underweighted numbers (weighted percentage).

**Table 2:** Differences in depression, suicidal ideation, suicidal plans, and suicidal attempts based on smartphone overdependence, stress, and covariates.

Variables	Categories	Depression				Suicidal Ideation			
		Yes	No	χ^2^	*p*	Yes	No	χ^2^	*p*
		*n* (%)	*n* (%)			*n* (%)	*n* (%)		
Independent variables									
Smartphone overdependence	Yes	2556 (36.8)	4354 (23.7)	427.82	<0.001	1173 (40.6)	5737 (25.5)	325.61	<0.001
	No	4544 (63.2)	14,533 (76.3)			1793 (59.4)	17,284 (74.5)		
Stress	High	4613 (65.1)	5150 (27.5)	1701.25	<0.001	2263 (76.2)	7500 (32.9)	1003.34	<0.001
	Some	2139 (30.1)	9182 (48.7)			612 (20.6)	10,709 (46.5)		
	No	348 (4.8)	4555 (23.8)			91 (3.2)	4812 (20.6)		
Covariates									
Biological factors									
Grade	1st	2244 (30.9)	6663 (34.4)	14.70	<0.001	926 (30.3)	7981 (33.9)	7.72	0.001
	2nd	2476 (34.4)	6431 (33.6)			1085 (36.1)	7822 (33.5)		
	3rd	2380 (34.7)	5793 (32.0)			955 (33.6)	7218 (32.6)		
Gender	Girls	4116 (57.7)	10,539 (55.8)	272.44	<0.001	1819 (60.3)	10,645 (46.3)	141.72	<0.001
	Boys	2984 (42.3)	8348 (44.2)			1147 (39.7)	12,376 (53.7)		
Social factors									
Residence area	Metropolis	3535 (51.2)	9592 (51.1)	0.01	0.992	1436 (49.8)	11,691 (51.3)	0.77	0.462
	Middle-sized cities	3110 (44.4)	8154 (44.5)			1337 (45.8)	9927 (44.3)		
	Rural area	455 (4.4)	1141 (4.4)			193 (4.4)	1403 (4.4)		
Socioeconomic status of household	High	2295 (33.3)	6406 (34.6)	71.67	<0.001	905 (31.7)	7796 (34.6)	55.53	<0.001
	Middle	3332 (47.1)	9770 (51.5)			1340 (45.8)	11,762 (50.9)		
	Low	1473 (19.6)	2711 (13.9)			721 (22.5)	3463 (14.5)		
Academic performance	High	1919 (27.1)	5811 (30.6)	48.24	<0.001	812 (27.5)	6918 (29.9)	37.72	<0.001
	Middle	2042 (29.3)	6105 (32.2)			781 (26.5)	7366 (32.1)		
	Low	3139 (43.6)	6971 (37.2)			1373 (46.0)	8737 (38.0)		
Psychosocial factors									
Perceived health status	Unhealthy	1138 (16.4)	1268 (6.8)	414.62	<0.001	667 (22.7)	1739 (7.7)	440.75	<0.001
	Fair	1976 (27.8)	3925 (21.1)			833 (28.3)	5068 (22.2)		
	Healthy	3986 (55.8)	13,694 (72.1)			1466 (49.0)	16,214 (70.1)		
Smoking	Yes	885 (11.9)	1197 (6.1)	234.49	<0.001	416 (13.3)	1666 (7.0)	158.51	<0.001
Drinking	No	6215 (88.1)	17,690 (93.9)			2550 (86.7)	21,355 (93.0)		
	Yes	1640 (22.4)	2632 (13.4)	288.40	<0.001	779 (25.6)	3493 (14.6)	230.08	<0.001
	No	5460 (77.6)	16,255 (86.6)			2187 (74.4)	19,528 (85.4)		
Drug use	Yes	146 (2.1)	93 (0.5)	145.88	<0.001	99 (3.2)	140 (0.7)	160.16	<0.001
	No	6954 (97.9)	18,794 (99.5)			2867 (96.8)	22,881 (99.3)		
Sexual intercourse	Yes	849 (11.6)	1084 (5.6)	322.17	<0.001	426 (14.1)	1507 (6.4)	257.04	<0.001
	No	6251 (88.4)	17,803 (94.4)			2540 (85.9)	21,514 (93.6)		
Physical activity	Sufficient	2486 (34.3)	6416 (33.2)	2.69	0.102	948 (32.0)	7954 (33.7)	3.57	0.060
	Insufficient	4614 (65.7)	12,471 (66.8)			2018 (68.0)	15,067 (66.3)		
Quality of sleep	Good	1191 (17.0)	5280 (28.0)	340.81	<0.001	416 (14.3)	6055 (26.4)	177.31	<0.001
	Moderate	2036 (28.3)	6684 (35.2)			833 (28.0)	7887 (34.0)		
	Poor	3873 (54.7)	6923 (36.8)			1717 (57.7)	9079 (39.6)		
**Variables**	**Categories**	**Suicidal Plans**				**Suicidal Attempts**			
		**Yes**	**No**	**χ^2^**	***p***	**Yes**	**No**	**χ^2^**	***p***
		***n*** **(%)**	***n*** **(%)**			***n*** **(%)**	***n*** **(%)**		
Independent variables									
Smartphone overdependence	Yes	331 (38.6)	6579 (26.9)	68.13	<0.001	195 (37.4)	6715 (27.1)	26.13	<0.001
	No	561 (61.4)	18,516 (73.1)			334 (62.6)	18,743 (72.9)		
Stress	High	671 (75.7)	9092 (36.5)	269.89	<0.001	414 (76.8)	9349 (37.0)	143.41	<0.001
	Some	172 (18.9)	11,149 (44.5)			93 (18.2)	11,228 (44.1)		
	No	49 (5.4)	4854 (19.0)			22 (5.0)	4881 (18.9)		
Covariates									
Biological factors									
Grade	1st	264 (28.4)	8643 (33.6)	5.49	0.005	155 (27.4)	8752 (33.6)	4.91	0.008
	2nd	327 (37.5)	8580 (33.7)			199 (38.3)	8708 (33.7)		
	3rd	301 (34.1)	7872 (32.7)			175 (34.3)	7998 (32.7)		
Gender	Girls	528 (58.4)	11,936 (47.5)	33.35	<0.001	342 (63.9)	12,122 (47.6)	59.24	<0.001
	Boys	364 (41.6)	13,159 (52.5)			187 (36.1)	13,336 (52.4)		
Social factors									
Residence area	Metropolis	441 (50.3)	12,686 (51.1)	0.19	0.828	287 (56.1)	12,840 (51.0)	3.28	0.039
	Middle-sized cities	398 (45.4)	10,866 (44.4)			216 (40.2)	11,048 (44.5)		
	Rural area	53 (4.3)	1543 (4.5)			26 (3.7)	1570 (4.5)		
Socioeconomic status of household	High	283 (33.6)	8418 (34.3)	30.34	<0.001	171 (33.8)	8530 (34.3)	23.77	<0.001
	Middle	369 (42.1)	12,733 (50.6)			205 (40.7)	12,897 (50.5)		
	Low	240 (24.3)	3944 (15.1)			153 (25.5)	4031 (15.2)		
Academic performance	High	223 (24.9)	7507 (29.8)	15.09	<0.001	132 (25.4)	7598 (29.7)	16.62	<0.001
	Middle	239 (27.4)	7908 (31.6)			124 (23.7)	8023 (31.6)		
	Low	430 (47.7)	9680 (38.6)			273 (50.9)	9837 (38.7)		
Psychosocial factors									
Perceived health status	Unhealthy	236 (26.4)	2170 (8.8)	167.85	<0.001	142 (26.7)	2264 (9.0)	108.60	<0.001
	Fair	233 (25.6)	5668 (22.9)			144 (27.1)	5757 (22.9)		
	Healthy	423 (48.0)	17,257 (68.3)			243 (46.2)	17,437 (68.1)		
Smoking	Yes	160 (17.8)	1922 (7.3)	129.09	<0.001	143 (25.6)	1939 (7.3)	276.58	<0.001
Drinking	No	732 (82.2)	23,173 (92.7)			386 (74.4)	23,519 (92.7)		
	Yes	271 (29.5)	4001 (15.4)	114.03	<0.001	205 (36.7)	4067 (15.4)	170.91	<0.001
	No	621 (70.5)	21,094 (84.6)			324 (63.3)	21,391 (84.6)		
Drug use	Yes	52 (5.7)	187 (0.8)	195.85	<0.001	40 (6.6)	199 (0.8)	187.71	<0.001
	No	840 (94.3)	24,908 (99.2)			489 (93.4)	25,259 (99.2)		
Sexual intercourse	Yes	182 (20.8)	1751 (6.8)	283.40	<0.001	151 (28.2)	1782 (6.8)	434.96	<0.001
	No	710 (79.2)	23,344 (93.2)			378 (71.8)	23,676 (93.2)		
Physical activity	Sufficient	328 (36.4)	8574 (33.4)	3.75	0.054	204 (39.4)	8698 (33.4)	9.11	0.003
	Insufficient	564 (63.6)	16,521 (66.6)			325 (60.6)	16,760 (66.6)		
Quality of sleep	Good	141 (16.6)	6330 (25.3)	53.51	<0.001	86 (16.5)	6385 (25.2)	24.95	<0.001
	Moderate	232 (25.4)	8488 (33.6)			138 (27.3)	8582 (33.4)		
	Poor	519 (58.0)	10,277 (41.1)			305 (56.2)	10,491 (41.4)		

Note. Values are presented as underweighted numbers (weighted percentage).

**Table 3:** Independent effects of smartphone overdependence and stress on depression, suicidal ideation, suicidal plans, and suicidal attempts.

Variables		Depression			Suicidal Ideation			Suicidal Plans			Suicidal Attempts		
		AOR	95% CI	*p*	AOR	95% CI	*p*	AOR	95% CI	*p*	AOR	95% CI	*p*
Smartphone overdependence	No	1			1			1			1		
	Yes	1.40	1.31–1.49	<0.001	1.44	1.32–1.57	<0.001	1.21	1.05–1.39	0.009	1.10	0.90–1.34	0.376
Stress	No	1			1			1			1		
	Some	2.72	2.44–3.05	<0.001	2.42	1.94–3.03	<0.001	1.38	0.98–1.95	0.067	1.38	0.84–2.29	0.205
	High	8.49	7.53–9.58	<0.001	9.84	7.93–12.21	<0.001	4.98	3.56–6.97	<0.001	4.87	2.94–8.06	<0.001

**Table 4:** Combined effects of smartphone overdependence and stress on depression, suicidal ideation, suicidal plans, and suicidal attempts.

Stress	Smartphone Overdependence	Depression			Suicidal Ideation			Suicidal Plans			Suicidal Attempts		
		AOR	95% CI	*p*	AOR	95% CI	*p*	AOR	95% CI	*p*	AOR	95% CI	*p*
No	No	1			1			1			1		
	Yes	1.09	0.81–1.48	0.569	0.83	0.49–1.43	0.508	0.65	0.29–1.46	0.296	1.45	0.51–4.11	0.484
Some	No	2.56	2.27–2.88	<0.001	2.10	1.64–2.68	<0.001	1.29	0.87–1.92	0.212	1.38	0.76–2.51	0.286
	Yes	3.71	3.23–4.27	<0.001	3.33	2.60–4.25	<0.001	1.37	0.91–2.07	0.132	1.86	0.99–3.50	0.054
High	No	8.06	7.08–9.17	<0.001	8.85	7.03–11.14	<0.001	4.36	3.01–6.31	<0.001	5.33	3.00–9.45	<0.001
	Yes	11.29	9.84–12.96	<0.001	12.58	9.97–15.88	<0.001	5.59	3.83–8.14	<0.001	5.47	3.09–9.68	<0.001

## Data Availability

Data were obtained from the Korea Disease Control and Prevention Agency (KDCA) and are available from https://www.kdca.go.kr/yhs/ (accessed on 2 January 2022).
